# The University and the Medical School
*An address to the Bristol Medico-Chirurgical Society.


**Published:** 1961-10

**Authors:** Philip Morris

**Affiliations:** Vice-Chancellor of the University of Bristol


### THE UNIVERSITY AND THE MEDICAL SCHOOL*

BY

SIR PHILIP MORRIS, K.C.M.G., C.B.E.

*Vice-Chancellor of the University of Bristol*

I have been wondering what led you to invite me to address you this evening and also what led me to accept your invitation. I found it difficult to provide satisfactory answers to either of the questions; and the second began to be important when I had to decide what to talk to you about. I am no expert in medical affairs. Indeed there is nothing in which you would be likely to be interested about which I could, as an expert, speak to you. The Society has been interested in the University ever since it began, and many of your members are closely associated with its work. I eventually came to the conclusion that my best course was to talk generally about the University and the place of the Medical School within it because they should here find a common interest. I was also led to a consciousness of the necessity to speak with due humility. The Medical School existed before the University and even before the University College which preceded it.

### EARLY HISTORY OF THE MEDICAL SCHOOL

Many of you will be familiar, some of you much more familiar than I am, with the early story of a Medical School in Bristol. There is evidence that some medical subjects were taught in Bristol in the Middle Ages, but the modern movement begins with the opening of the Bristol Medical School in 1833. There was at about the same time a rash of provincial medical schools. The original object of the School was to provide some systematic instruction for groups of students attached to already existing hospitals which were, notably in the case of the Infirmary, of much older foundation. In its early years, the School obviously met a need and succeeded; and it quickly acquired a high reputation. It had no endowment and was poorly provided with resources, and these deficiencies had their effect in due course. In the later years of the century a Medical School entirely dependent upon fees paid by the students was already becoming an anachronism and things were not going so well. It was, however, active enough and enjoyed a high enough local reputation to play its part in the foundation of a University College. Since then the fortunes of the Medical School have been interlinked with those first of the University College and, since 1909, of the University. But I must avoid, what is far from my intention, developing a historical theme. I prefer on this occasion to speak more discontinuously, and to mention some things which are of current interest, and perhaps a few of more fundamental importance. To add interest to what might otherwise be a bald and unconvincing talk, I prevailed upon the Bursar to display a model or two and also some drawings of the new Medical School which is rapidly taking shape on the side of St. Michael's Hill. I am very grateful to him, as I am sure you will be. A casual look at what he has put out for us to see shows that things are very much on the move all around us. I thought I would devote a few minutes to putting the considerable activities of the present time into some sort of perspective.

The story of the early years of the Medical School is an integral part of the prenatal history of the University. The gestatory period lasted until 1909 when a new life began. The Medical School had been fully incorporated into the University

*An address to the Bristol Medico-Chirurgical Society.

College almost twenty years earlier, and a College Faculty of Medicine was already in existence. There was no abrupt change in the arrangements as a result of the Charter, but the fact that a University had come into existence with the power to grant its own medical degrees helped to knit together the very divided and dispersed medical teaching going on in the City. Before the University began, clinical teaching had remained largely independent of the University College. For some years, the students completed their pre-clinical work and then chose between the Royal Infirmary and the General Hospital as the place for their clinical studies. Rumour has it that one of these was very much better placed than the other, but it does not reveal which it was. It was not until a few years after the termination of the first world war that the medical curriculum came completely under University management, and it was not until 1940 that the amalgamation of the Infirmary and the Hospital completed the process of unification. It was fortunate that this unification was achieved well in advance of the establishment of the Health Service and the story of medical education and of the Hospitals associated with it might have been different had this process of unification been successfully achieved, as it might have been, very much sooner.

### GROWTH OF THE UNIVERSITY

But following the story of medical education has caused me to run ahead of myself. It is now difficult to appreciate how short a period there was between the establishment of the University in 1909 and the beginning of the first world war. Certainly the infancy of the University was attended by many difficulties. It stood in great need of accommodation for its work and all facilities appropriate to a University. Building activities were necessarily interrupted by the first war and the Main Building was not available for use until 1924. Thus the first period of real growth and development extends from the end of the first world war to the end of the second. During that time steady progress was made with the provision of facilities both academic and residential; and the University began to be accepted within the family of universities as an academic centre. All the plans and ideas which were being put into action followed the general principle of developing a small and intimate collegiate University. In the last session before the second world war, the total student body was round about one thousand, and staff little more than a hundred. The University was well known for its friendliness and intimacy and in some academic fields it had more than simply begun to make its mark. The Medical School had in a wide variety of ways integrated itself with the general life and government of the University, and this was greatly to its advantage because it was still small and by no means all candidates who presented themselves for and successfully gained admission were well prepared for the studies that they wished to pursue. The second war broke out at a most unpropitious moment in the life of the University, and many of those who were then involved in its affairs must have wondered how the threads would be picked up and life resumed when the war eventually came to an end. As the University buildings were seriously affected by war damage, it would be forgivable if people had allowed their faith to sink to a low ebb. This, however, did not happen as the vitality which immediately began to show itself at the conclusion of the war showed.

The third convenient period in the story both of the University and of the Medical School relates to the years between the end of the second war and the celebration of the Jubilee in 1959. For good or ill, between those years the whole scene both in relation to the University and the Medical School has changed, and considering the position as we do in the beginning of 1961, we have to note that the whole scene is beginning to change again. During this period, growth and development have been constantly with us. Indeed, there are many who think that there has been too much of both. However that may be, the student body rose to over three thousand, and is for the time being settled in that region. The additional numbers have not happened as a result simply of the further development of existing activities. It has fortunately been possible for the variety of work done to grow as the size of the student body has increased—and not incommensurately with it. Thus the sources from which the strength and vitality of the University derive are many and varied and opportunities for inter-action between different fields of study and different kinds of student have increased as the University has become inevitably a large-scale—as opposed to a small and intimate—affair. In such a period there are always dangers that the real purpose of a University will become submerged in anxiety and over-activity about the ways and means of development and growth. I feel sure that in this University this has not happened and the credit for this is very largely due to many people who have given long and faithful service to the University over many years under very varying conditions. Looking back over this period, one too easily forgets the extreme difficulties of making good the ravages of war. Indeed, we still have ruins to remind us of the war although happily the most grievous loss which the University has suffered is now slowly but surely being made good. The Great Hall—which incidentally I myself never saw in its original condition—is being worthily restored, and such beauty as it has will be very much due to the generosity of a member of the family of the original donor of the Main Building who, with rare foresight, enabled the University to buy the necessary oak immediately after the conclusion of the war. This having been well seasoned is now being worked and installed. Sometime in 1962 this will be finished. Many of us will then be able to see for the first time what it used to look like. Although there was much work to do and much money had to be spent in order to accommodate the increased numbers, the surprising thing on the whole is that the University should have grown to three thousand students without the completion of any major building except Queen's Building. Those who originally founded and developed the University must have been gifted with unusual foresight because their work turned out to be versatile and usable in very different conditions. During the same period the Medical School established itself on a steady level equivalent to rather more than twice its pre-war size with the majority of its students beginning their University work having completed the first M.B. beforehand. But during the period while increased facilities were provided for the Medical School in a number of ways, it did not prove possible to embark upon the two things which were most required, namely the provision of a new Medical School designed for its purpose and adequate to its increased size on the one hand, and the re-building of the Teaching Hospital on the other. Both of these objectives remain to be achieved during the period upon which the University and the Medical School have already begun.

### THE UNIVERSITY TODAY

The size of the University is for the time being stabilized at about three thousand five hundred students, but there are many deficiencies to be made good. Remedying some of the present deficiencies will inevitably make some expansion in the total size of the University follow as a consequence. Further, the demand in the country for a more generous provision for university education is strong, and successive Governments have felt that the country must look to its higher education, and particularly to the scale of it, if it is to maintain its place in the modern world. In conditions as we know them today, it seems inevitable that universities should be actively on the move, and any relaxation into isolation and detached contemplation seems to be out of the question. However this may be, the University has a very substantial programme of development actually in operation, and a still bigger one in contemplation. Indeed, it is a sobering thought that all the extensions already planned and under active consideration are in scope and scale a good deal more extensive than the University as we know it today.

The expansion of the University is no simple matter. It is not simply a question of those who want laboratories, lecture rooms and the like sitting down and working out what they would like to have, although all this has to be done and is very important. But it has to be remembered that the University is already a large community, and the consequences which follow from making it a community of twice the size raise many problems. It would, for example, be a very great tragedy if the quality of student life deteriorated through an undue concentration in purely academic requirements to the exclusion of facilities for living, feeding and informal association. It is therefore absolutely necessary that any expansion programme should be well balanced, providing in as reasonable a manner as possible for all the needs of a very much larger community. I will not at present enter into the complexities of maintaining a balance between the accommodation available in the various subjects. A moment's reflection will make it plain that even the academic requirements of undergraduates, because of the variety of subject which seems to be maintained, give rise to intricate and difficult problems. Also, those who know most about University life and University people would be the first to accept that the dispersed and rather loosely constructed machinery of government in the University is not ideal for the purposes of devising and carrying into effect a carefully co-ordinated long-term plan.

I think I should spend a few minutes reciting the list of projects actually being carried out and those in contemplation, so that the scale and diversity of all that is going on may be apparent. In addition to the re-building of the Great Hall as a very proper act of piety, the main Library is being extended to its originally designed size, and all the administrative staff and offices will be removed from the Main Building when the new Senate House, now in course of erection on what used to be known as the Hiatt Baker Garden, is completed. What is now known as the Main Building will then accommodate the Faculties of Arts and Law and the Library, but will not be by itself sufficient for these purposes. Consequently, a large building in the south-east corner of Berkeley Square is in course of erection in which will be accommodated the Departments of Economics, English, Spanish, and the staff of the School of Management Studies. The new Medical School, as everyone present will know, is rapidly taking shape and all the pre-clinical Departments will move into the first stage as soon as it is completed, thus liberating accommodation for the Faculty of Science. The demands of the Faculty of Science cannot however be met without further additional building, and work has begun on a large new School of Chemistry, comparable in size to Queen's Building, on the slope of St. Michael's Hill below Queen's Building and the new Medical School. Also the building of the Physics Laboratory is to be completed and its unfinished end, which has for so long disfigured it, will soon disappear. Incidentally, in the course of completing the Physics Building, a substantial piece of work in connection with an adequate and satisfactory water supply to the whole area will be carried out. While all these buildings when completed will both solve many problems and also create others, they do not effectively provide for the requirements of the Departments of Mathematics and Education, and some further projects will have to be devised to do so. The completion of all these buildings will inevitably bring a big increase in the undergraduate population, and a new Union in time for such an increase in undergraduate numbers becomes almost a first priority. A project for a new Union has been planned and the commencement of work upon it is unlikely to be long delayed. The expansion which is going to happen means inevitably that the desire to accommodate the majority of students in Halls of Residence has to be for the time being set aside in favour of a more modest objective of maintaining the same *proportion* of students in Halls of Residence. To achieve this more limited objective means a very substantial programme of residential building which is making good progress and will, we hope, make even better progress in the near future. Churchill Hall already completed, Clifton Hill House rapidly nearing completion, an extension to Wills Hall about to begin, and four new men's Halls in the Wills Hall area, two to begin quite shortly and two to follow, comprise the present definite programme. There will still be available space for a further extensive programme later on. The position can be put quite shortly something like this. Additional academic accommodation brings more students; more students need more staff; all members of the University need somewhere to live and senior common rooms and Unions in which to get to know each other. Expansion depends upon the provision of accommodation and building operations depend chiefly upon the rate of the flow of the money with which to pay for them.

### THE NEW MEDICAL SCHOOL

I do not propose to speak in detail about the new Medical School which is now being built. The model and the drawings give a better idea of it than I could possibly give in words. Shortly put, it represents a decision on the part of those concerned to provide adequately for a Medical School of about the present size in a manner which integrates the Medical School with the University on one side and the proposed new buildings of the Teaching Hospital on the other. What this project means is perhaps of greater significance and importance than its physical details. It represents and gives effect to some very important and fundamental decisions of which the following are examples: that a Medical School should be part of the University and integrated with the University to the maximum possible extent; that the clinical work of the Medical School should be conducted in a Teaching Hospital; that the University and the Medical School should be geographically close to each other; that the administration of the Teaching Hospital as part of the Health Service on the one hand, and the administration of the Medical School by the University as an autonomous corporation on the other can proceed in harmony with each other; and that all the people concerned, whether they think of themselves as belonging to the University or the Hospital, should accept and understand a loyalty to an objective which transcends both. I could add substantially to this list, but I have said enough to emphasize the responsibility which everybody, and in relation to the present meeting, especially the members of the medical profession, carries for the success of what is being done. The only aspect of this complex of problems which I want to develop for a few minutes now is whether a Medical School is rightly placed in a University.

### MEDICAL EDUCATION

A Medical School is a social institution and its essential function is to ensure that the need of society for the continuance of the practice of medicine and the prosecution of study and research in fields of knowledge relevant to it is satisfactorily met. At first sight, it might seem that the importance of professional training was such that a completely independent institution solely devoted to the requirements of the profession might satisfactorily meet the need. But it has to be remembered that the practice of medicine is an art based upon the application of knowledge derived from many fields covering a wide variety of subjects. The medical practitioner has never been and can never become merely an instrument for the application of ascertained knowledge to particular conditions and situations. The essence of the medical practitioner's art is that he must consider each patient as a whole person, and in treating his patients, rely to the maximum possible extent on ascertained knowledge where that is sufficient for the occasion. While, therefore, the training of the doctor and the education of the student as a person must always remain intermingled, it is possible and useful to distinguish between them. The knowledge which the medical profession must have at its disposal is derived from so many fields of study that it ought to be studied and advanced in circumstances in which it can rely upon and interact with all those fields of study which are being pursued in their own right. By finding itself in company with schools concerned with a wide variety of fields of study, within a University, a Medical School finds the right conditions for its wellbeing and advancement. Like other schools within a University, it needs a sufficient measure of autonomy; but also, like other schools, it gains rather than loses by accepting the discipline and benefits of sharing with other schools a responsibility for the maintenance and advancement of knowledge as a whole. So far as the training of students in preparation for the practice of medicine is concerned, it is clear that those who wish to become doctors must be educated in the ways and habits and customs of their fellow men and women as much as in the scientific knowledge which they hope to have at their command. Thus there is advantage rather than loss in the mingling of medical students with other students in the study of any relevant subjects in which such a mingling of students is practicable. The knowledge which the medical student needs to acquire is not different from that which others also need simply because he is proposing in due course to apply it in a special and particular manner. Further, as the advancement of medicine depends both upon the development of its practice as an art and also upon the advancement of knowledge upon which the practice of the art relies, the foundation of progress is to be found not only in departments devoted to medical subjects but also in a wide variety of other subjects also. The chief arguments, therefore, for placing a Medical School within a University are that it finds there the right conditions for its own healthy growth, and can in such circumstances make its own contribution to other disciplines. The greatest possible inter-action between disciplines which is compatible with good and intelligent government and administration is to the advantage both of the studies themselves and also of society as a whole. Perhaps this is a question which does not need to be argued. Nevertheless, it is a good thing to examine the reasons for a state of affairs which exists and which is at present in process of very substantial development.

### THE MEDICAL CURRICULUM

What I have been saying tempts me to trespass upon ground which some think should be sacred to members of the medical profession, and to refer to the present state of the medical curriculum. I must be careful because in everything I say and in most things that I think, I am constrained by fear of colleagues both in the University and in the Teaching Hospital. I maintain quite definitely that those who are not medical practitioners have a contribution to make and a function to fulfil in relation to medical education. My reasons for holding this view will be apparent from what I have already said about the appropriateness of a Medical School finding itself within a University. Indeed, the very fact that a Medical School is properly placed within a University suggests that it cannot live its own life free from the influence of others and from the consequences which flow from co-operation and inter-action. In any event, the few things I would like to say and the questions that I want to raise will not perhaps turn out to be very controversial after all. It is first of all necessary to ask what kind of students a medical school wishes to admit, and by what criteria they should be selected. In this context it should be remembered that it is only during comparatively recent years that the first M.B. has been taken and passed before a university course begins. Here in Bristol students were being admitted some to take an intermediate course and others to embark immediately upon a course leading to the second M.B., as recently as since the end of the last war. Indeed, it is still not impossible for students intending subsequently to take a medical course virtually to begin their scientific education in the University. But the tendency towards the admission of students with the first M.B. as an entrance qualification has increased. The result is that the majority of students who are admitted have been concentrating upon scientific subjects during their concluding years at school. What is happening in practice would suggest that the best educational preparation at school for a medical course was one with a stronger scientific bent. It might even seem to suggest that students for admission to a medical school should be preferred on account of their scientific ability and attainments. There seems to be no doubt that in the present state of medical knowledge, medical students must have a sufficient scientific grounding; the important question to keep under consideration is exactly how much weight should be attached to ability, and attainments measured by examinations, in scientific subjects.

I do not attempt to answer this question but rather content myself with asking it. I prefer to go on to raise the problem which has been caused by the exceptionally rapid developments which have taken place in the medical field during recent years. It is difficult to speak in a few words about the nature of these changes because they are so various and cover such a wide field. It is nevertheless possible to point to inter-related developments which are causes, perhaps the chief causes, of the changes which have come about. The first important development must be looked for outside the specifically medical field because it is to be found in the great advances which have been made by what I would call physical and biological scientists. The story of these advances affords an illuminating example of the profitable results of inter-action between different disciplines. The development and elaboration of techniques has been assisted by as well as itself helping to bring about a greater understanding of natural phenomena. The second important development has been the rapid adoption within the medical field of methods and techniques which have their origins elsewhere. The effects upon all those involved in the treatment of patients have been far-reaching. As in the biological and physical sciences, fragmentation and specialization have increased with the advancement of knowledge, so in both the study of medical problems and in the application of new knowledge and techniques to the treatment of patients, a similar tendency towards specialization and fragmentation has asserted itself. It is easy to criticize this tendency because of the difficulties and complications which follow in its train. Equally, the logic of the situation forces us to the conclusion that the sheer quantity and elaborateness of new knowledge and new techniques makes specialization quite inescapable. In fact, the acceptance of specialization is part of the price that has to be paid for both the advancement of knowledge and a speedy application of new knowledge to current clinical problems. If this price must inevitably be paid, the important question for those engaged in medical education to ask is how a curriculum can be devised which does not lose in the bye-ways of specialized fragments the unity which is so essential to the student.

There is no easy answer to the very difficult questions which arise in the course of attempting to reform the medical curriculum. Indeed, it is only when the attempt is seriously initiated that the many real difficulties begin to be appreciated. Despite all the difficulties, it is very necessary that all efforts should be bent towards making some progress in reforming the curriculum because time is not on our side, and the advancement of medical knowledge should not be arrested or hampered because of avoidable weaknesses in the education of a rising generation. It has already become impossible, even with the assistance of years of postgraduate education and training, for any single doctor to be a master of all practising specialties or of all aspects of medical science. The question therefore becomes whether it is possible to identify and systematize what is fundamentally necessary as a grounding in the study and practice of medicine, or whether the best that can be done is to continue to include in a medical course elements of all the relevant specialties and subjects. However this question may be answered by those who have the chief responsibility in this matter, there is one important aspect of this problem which it is dangerous to overlook. Doctors at present, and still more in the future, whatever the nature of their employment, have powerful positive instruments at their disposal. These positive instruments not only require a sound intellectual discipline for their understanding but they need at the same time to be seen in a rich and varied medical context which is changing all the time. The old-fashioned idea that for work of high quality, a trained mind was a necessary pre-condition is strengthened rather than weakened by the present and any future state of affairs. A newer conviction that the trained mind must not work in a vacuum but in the varied contemporary setting in which it finds itself is also of great importance. However the medical curriculum may be arranged, it seems to need to be tested by two criteria; namely, whether it will train minds by including sufficient study in depth, and also whether it will provide an accurate and informed view of the general state of medical affairs combined with a real sense of perspective. But there are other difficulties also to be encountered. The suitability and effectiveness of a course of medical education can be judged only in relation to those for whom it is intended and their ability to pursue it; and ability to pursue it means far more than is usually meant by a minimum standard of ability and attainments.

There is one aspect of the reform of medical education which is of such importance that it calls for some consideration by itself. The aspect to which I refer is implicit in the description of the present state of affairs and what was happening to change it. Some of the elements necessary to the medical course lie more on the side of academic training and find themselves more appropriately placed in university departments, while others are of a clinical nature, having to do with the application of knowledge to the treatment of patients as well as with training in the very deep and complex personal relationships which must subsist between doctors and patients and find themselves necessarily in a clinical setting, namely in a hospital. Now the primary purpose of a university is teaching and research seen as complementary activities. I suggest that the primary purpose of a teaching hospital is the treatment of patients and research into the cause and cure of disease similarly seen as complementary activities. Unless knowledge advances, teaching mortifies; and it could be the case that unless knowledge of causes and cures advances, treatment, and more important still, training in diagnosis and therapeutics, loses its vitality. While the university on the one hand and teaching hospitals on the other are different institutions with different purposes, they must in relation to a medical school act in full co-operation based upon a common conviction that the final outcome of the efforts of both depends upon people rather than simply upon knowledge and facilities, and upon the adequacy of people to turn things and facilities to the greatest advantage. This all seems, as stated in this relatively simple manner, very obvious. Further reflection, for which there is not now time or opportunity, will readily suggest the kind of difficulties with which those who bear responsibility in hospitals on the one hand and in universities on the other are confronted. We are indeed fortunate here in the general attitude and spirit which is brought to bear upon the solution of some of these problems, and it is enough to say at the moment that the big building projects in hand or in immediate contemplation for the Medical School, Hospital and Dental Hospital provide both novel problems and new and increased opportunities.

### CONCLUSION

Universities will fail to make their full contribution to society if, either in relation to medicine or in relation to any other profession, they allow themselves to become schools of professional training. The final arbiter of academic questions for a university is the extent to which knowledge in any field is advanced in its own right and irrespective of its consequences. The final arbiter in the case of a hospital and of the medical profession is whether or not everything that is humanly possible is done for patients in need. If there is conflict here, and perhaps it is more apparent than real, the resolution of it is the greatest of contemporary challenges. The success with which we meet this challenge depends upon our hearts as well as upon our heads, as everything depends upon intelligent and selfless co-operation between individuals. Big demands are made and some sacrifice is required. New opportunities are in course of presenting themselves and it was for this reason that I thought I could at this juncture accept your invitation and speak to you rather discontinuously and discursively about matters in which, I take it, we are all, in our various ways, interested and concerned.

